# Strangulated Hernia, Treated by Opium

**Published:** 1860-04

**Authors:** S. W. Noble

**Affiliations:** Le Roy, Ill.


THE CHICAGO

### MEDICAL JOURNAL.

VOL. III. APRIL, 1860. NO. 4.

### Original Communications.

### ARTICLE 1.

### STRANGULATED HERNIA, TREATED BY OPIUM.

READ BEFORE McLEAN COUNTY MEDICAL SOCIETY, OCTOBER 1857, BY S. W. NOBLE, M. D., AND PUBLISHED BY RESOLUTION OF THE SOCIETY.

Gentlemen: When I received an appointment at your hands to read an essay on this occasion, I determined to write on some subject relating particularly to the practice of our profession. And while contemplating in my own mind what subject to select for discussion, I was called to treat a case of Strangulated Hernia, which reminded me of some experience I have had in the treatment of that disease which I have long thought of presenting to the consideration of this Society.

I hope, therefore, no apology will be necessary for departing from the course of my predecessors on such occasions, and presenting a simple relation of facts, as I have observed them, in the treatment of a few cases of Strangulated Hernia. It is not expected that we will go into a full description of the nature and causes of Hernia in a paper of this kind; neither is it necessary, for the disease is so plainly described in every work on surgery, that it is expected of every practitioner that he will recognize the presence of a Hernia so soon as it becomes strangulated.

What I propose, then, for discussion to-day, is the indica-cations of cure which present themselves in the treatment of a case of Strangulated Hernia, and the means by which these indications may be fulfilled.

The first and principal indication of cure is to relieve the stricture and return the Hernia, which is to be accomplished, if possible, by taxis, as directed in the books. But if the taxis, aided by reliable auxillaries, fail, I believe that all authors, so far as I have read, lay it down as a rule, and urge the importance of the practice, that as soon as the surgeon is satisfied that the strangulation cannot be relieved by the taxis, he is bound to operate, and that without delay, as the only chance of relief. And the great majority of experienced surgeons agree that in regard to this operation, error is more frequently on the side of delay than of precipitancy.

"Two circumstances," says authority, "demand its instant performance: a conviction that by no other means than the edge of the knife, directly applied, can the abdominal outlet be so enlarged as to relieve the constriction and admit of replacement; also a conviction that already inflammatory action has advanced so far that either ulceration or sloughing is inevitable in the protruding parts. In the one case they operate to relieve the stricture—effect replacement hoping to arrest inflammation. In the other they operate to relieve the stricture, and leave the hernia unreduced, and prevent fatal extravisation of intestinal contents within the abdomen."

In the face of the above, and long established convictions, a man would be considered reckless indeed, who would for one moment hesitate to operate, or would try other and milder means to produce the same effect. Were it not for the old maxim: "That the true science of surgery consists in relieving surgical cases without the use of the knife," I should not presume to offer any objections to knifing a hernia; neither will I attempt to offer a substitute for the knife in all cases of strangulation. But while the watchword of the age is progress, I don't think it impossible for us to make some change, at least, if not improvement, in this long established course of practice.

And when we take into consideration the mortality attending the operation, which is admitted on all hands to be great, it is not to be wondered at that we should hail with pleasure any means calculated to reduce the amount of mortality from that cause. This is particularly interesting when we reflect for a moment on the frequency of the accident or disease. I have seen it estimated by some writer in a late journal, that one out of every fourteen persons in the community have a hernia. This proportion, when viewed in connection with the universality of the disease, would show that a larger number of the human family are affected with it than with any other disease that flesh is heir to.

The common disorders of life are, for the most part, limited to particular portions of the earth's surface; some limited to particular seasons, some to particular races, some to particular classes of the race, and some to certain periods of life; but hernia is not subject to any of these limitations. The American and the Asiatic—the infant and the octogenarian—the citizen and the backwoodsman, are all alike subject to it. It may be said of the unfortunate class so affected, that they stand in jeopardy every hour; they know not but the next step or cough, or slight irregularity will induce strangulation that shall prove fatal or otherwise, according as the physician in attendance may happen to be skillful or otherwise.

The treatment that we propose for the relief of Strangulated Hernia is full and oft repeated doses of opium until complete narcosis is produced. When given in this way opium will evidently produce a more complete relaxation of the muscular system than any other known remedy. It possesses this advantage over most other relaxing medicines: you can manipulate without giving pain. But, on the other hand, if you relax the system perfectly with tartar emetic or tobacco and apply the taxis, you cause pain in the parts which excite contraction in the muscles implicated in the hernia, which are the very ones you wish to relax. The same objection applies to chloroform, with this slight exception: the patient is not conscious of the pain, and you have not the influence of volition to contend with.

Opium, likewise, when given in large doses; say from 3 to 5 grains to an adult; arrests vomiting, which is one of the most unpleasant symptoms, quiets the irritability of the system, and effectually prevents inflammation and mortification in the strangulated portion of the bowel. When the patient is thoroughly and permanently under the influence of opium, if the stricture is not relieved and the bowel returned spontaneously, it may be readily accomplished by a renewal of the taxis. At least that has been my experience in these cases.

I have not arrived at these conclusions through the channel of any fine spun theory, but have given this subject much thought and study, and if, after hearing the result of the cases treated in this way, which I am now going to report, the members present are satisfied that I am in error in recommending this course, or that the practice would be unsafe and empirical, and will show satisfactory grounds on which they base their convictions, I will agree, in the next case of Strangulated Hernia that I treat, to send for a surgeon, early. But while I am in the same state of mind I am to-day, I shall never resort to the knife to relieve stricture in those cases.

My thoughts were first directed to this subject strictly by accident, though I have since seen in some of the journals (that I have been unable to lay my hand on while preparing this paper) that an English surgeon recommended the opium treatment in cases of irreduceable hernia, to fill the same indications.

I will now give a report of the first case treated in this way, which will fully explain why I first ventured to try the opium treatment, while the result of the case will be ample excuse for trying it in the next case.

Called, March 7th, 1850, at 4 P. M., to see H. B., (known to most of you,) whose hernia had become strangulated about noon of the same day, which caused severe pain in the bowels, constant retching and vomiting of stercoraceous matter—in fact, all the symptoms of Strangulated Hernia in their aggravated form, which had not been relieved at all by an irregular, who had been treating him for the two hours previous to my visit, as the "most desperate case of cholera morbus he had ever witnessed in a thirty years' practice." I placed the patient in position, and applied gentle taxis for a few minutes without relief. I then gave nauseating medicines to relax the muscular system and applied cold to the tumor, with no better effect.

Being fresh from the schools and fully impressed with the importance of an early operation, I very candidly informed the patient and his family that there was no hope of relief without an operation, and urged the propriety of its immediate performance, after fully explaining the danger attending the operation.

The old gentleman readily consented to submit to whatever course I thought best, but was inclined to think I was too young to use the knife with safety and proposed sending to Bloomington for an old physician. I expostulated with him for awhile, giving it as my opinion that, owing to the condition of the roads, which were almost impassible at that hour of the night, it would be too late for council to be of any avail in his case before he could procure it. Seeing, however, that he was determided in his course, a messenger was sent in haste for Dr. Thomas, who was at that time supposed to be the oldest physician in Bloomington. The night being unpleasant, Dr. Thomas refused to turn out, but told the messenger to hurry home and tell me to give the patient a big dose of morphine. By the way, we would here remark that we have since learned, although we did not know it at the time, that the Dr. intended his morphine as a curative instead of a palliative. That he has treated hernia in this way. The messenger being satisfied that there would have to be an operation performed, was not willing to return without council, so he called on Drs. Colburn & Rogers (our venerable President) who were soon enlisted in the spirit of the case and gathered up their knives, a bottle of ether and a copy of Gibbons' Surgery, and were at the seat of action by 2 o'clock A. M., when the same course of manipulation was again gone through with, after relaxing the system thoroughly with an injection of tobacco, without being able to reduce the hernia. I had given a pill of opium when the messenger started, which had quieted the irritability of the stomach for a time, but the effect had now entirely worn off, and the symptoms continued severe, with a decided increase of pain in the tumor.

The patient and friends were again assured that there was no hope of relief without an operation, which was again explained to him, and the dangers frankly admitted. Consent to an operation was given.

Day was dawning, and as we were all tired from our night's exercises, we agreed to take breakfast, which was nearly ready, and arrange matters for operating as soon as it was light. After breakfast we cut our adhesive strips; threaded our needles; constructed an operating table; got the patient on it, and partly under the influence of ether. I had the tumor in one hand and the knife in the other, just ready to distinguish myself as a surgeon, when the patient cries, "Hold! I fear I may die, and should like to see my wife once more." We tried in vain to persuade him out of this notion, but he plead so strong we finally agreed he might have a parting word with her. Now it is to be regretted that these old people did not live very happily together, hence it is not to be wondered that when the old lady was ushered into his presence she did not show signs of the most heartfelt sorrow at the prospect of being so soon left a widow. Be this as it may, when he saw her, says he: "Stop! take away your knife; I will die and be out of her way." With this he crawled from the table, and re-arranged his bed, and all we could say or do we were unable to persuade him back to the table. We plead with him as we would for his life, believing as we did at the time, there was no salvation for him without the knife. We likewise thought there was a strong probabiliy of saving him by an operation. Finally we gave it up. He said that he wished to die, and all he wanted of us was to give him something to die easy on.

After a very grave consultation, it was agreed to give him morphine enough to relieve him of all suffering this side of the grave. We gave a large dose, something over a grain. We then took a very feeling farewell of our old friend, and Dr. Rogers administered what spiritual consolation he could under the circumstances, and left for our several homes. He took his morphine, turned over and went calmly to sleep. In the evening, about 5 o'clock, I was passing the house, and seeing no bedstead sitting out, or other signs of a funeral, I ventured in to see how he was doing. I found him lying on his back, presenting more the appearance of a cadaver than any thing else, breathing about eight times per minute, pulse forty, full and regular, skin covered with a cold, clammy sweat, muscular system perfectly relaxed. I thought from appearances he would die, and that soon, from the effects of the morphine, if from nothing else. But I concluded that I would again try to reduce the hernia, when, to my utter astonishment, there was no obstacle at all in the way of replacing the bowel. I recommended the cold douche and strong coffee, to relieve the system from the effects of the morphine, but still thought he must die sooner or later from gangrene, according to the books, judging from the length of time the hernia had been strangulated, so I did not venture to call upon him again for two days, when I found him sitting up quite comfortable, and very much inclined to laugh at the uncertainty of human prognostications.

He convalesced rapidly, and lived to give his family and the county authorities no little trouble.

Case II.—The next case of hernia that I saw was in 1852, in a boy 2½ years old. Had been troubled with a scrotal hernia for six months. The patient was much reduced by a protracted attack of ague. His hernia became strangulated and was treated three hours as a cholic; but owing to the severity of the symptoms, and the fact of his throwing up fœces, the parents became alarmed, and I was called in some four hours from the onset. I thought it was an unfavorable case for an operation; besides, by this time I had myself pretty well persuaded that the opium treatment was safe and reliable. So I concluded, after an ineffectual effort to replace the bowel by taxis, to give the opium a fair trial in this case. Accordingly I prescribed opii. gr. 1, every two hours, until he was thoroughly narcotized. Being fatigued, I laid down with directions for them to call me as soon as he slept soundly and could be moved about without waking. His stomach retained the medicine, and I was not called until after he had taken the third powder, when, to my surprise, on examination, I found the hernia had entirely disappeared. Patient slept till morning, when his bowels were moved by enema. Quinine was prescribed for his ague, and a truss supplied for the hernia, but the ring was so sore from our manipulations that he could not wear the truss. Strange to say, the bowel has never descended since, and to all appearance the hernia is radically cured, though we do not attribute the radical part of the cure to the opium.

Case III.—Though not successful, served to convince me more fully of the importance and reliability of the opium. I report it cheerfully, notwithstanding it is very uncommon for physicians to report unsuccessful cases:

June 1st, 1854. My friend Dr. Craig requested me to visit a patient of his whose hernia had become strangulated about six hours previous to my visit. The Doctor had tried the taxis, aided, I believe, by the whole list of auxillaries, without being able to reduce the tumor. The patient *pointedly* refused to submit to an operation. I then suggested the opium treatment, which the Doctor readily consented to, and we ordered ½ gr. morphine, with a little soda, every hour, until narcosis was produced, when the Doctor was to be notified. But unfortunately for science and the man's own welfare, the family were not strictly honest with us, and when the man began to roll up his eyes and see sights, they put the powders aside, and told Dr. Craig, on his next visit, that the patient had taken them all, but had thrown them up. The Doctor repeated the prescription with the same relief to the pain and vomiting, but was unable to narcotise him thoroughly for two days, when business called him from home. He left orders with the family for them to send for me immediately; but they were so strongly prejudiced against the use of opium, and the man was resting so easily, that I was not sent for until late in the evening of the 25th, when there was a return of all the symptoms, with a decided increase of pain and soreness in the tumor. I prescribed morphine, gr. 1, every hour, and calculated to stay and see why it was that he would not become narcotised, but was called away and detained until morning. When I returned I found he had rested well, and they reported him to have taken three doses of the prescription. This I doubted, and directed 1 gr. morphine every hour, and stayed to see that they were given.

In about one hour after he took the second powder I succeeded in reducing the hernia without any trouble. He drank a strong cup of coffee and got up to the stool and had a very copious evacuation from his bowels, but died that evening, evidently from gangrene, though there was no post mortem allowed.

This man's life, I verily believe, might have been saved if they had been more honest with the Doctors.

[Cases IV, V, and VI, were all cases of Inguinal Hernia, in male adults, relieved by the taxis within eight hours from the first prescription of opium. The treatment and results were so near like cases I and II, that we deem it unnecessary to cumber the pages of your journal with an extended report.—Sect.]

Case VII is one of unusual interest, from the length of time the stricture remained, the amount of opium taken, and the favorable termination. It shows in an eminent degree, the power of opium in preventing inflammation and mortification. It likewise demonstrates what a degree of tolerance for opium is established or created by severe pain. One gr. of opium is a full dose for the patient under ordinary circumstances, yet under the influence of pain he took, in thirty-six hours, seventy-two gr. with impunity.

Called, July 22d, 1857, to see G. D. C., but being indisposed at the time, my friend, Dr. I. M. Coleman, kindly consented to visit him for me. He found a large scrotal hernia strangulated. After trying all the usual remedies and manipulations without being able to relieve the stricture, at my suggestion he put him on opium, gr. ii. every hour. He took the prescription all night, but owing to the severe pain and irritability of the stomach, (some of the opium being thrown up, no doubt,) he was not narcotised. Prescription was continued twenty-four hours longer without any perceptible effect on the system except the partial relief of pain and soreness. Vomiting was frequent, but less stercoraceous. I was now (the 23d) able to visit the patient, and Dr. Stipp, of Bloomington, was added to the consultation. Dr. Stipp was in favor of an operation but thought it too late to be of service. At his suggestion we introduced a long flexible tube, and filled the rectum and colon with warm water, which passed away without fœces. We then injected laudanum up the rectum, but it was not retained. The case now was considered almost hopeless, but I was anxious to try the opium further, so we agreed to give him sulp. of morphine, gr. 1, every hour, until he was narcotised. His stomach rejected the first dose, when a second was given immediately, and I remained with the patient to watch the effect. After taking the fifth portion of morphine, the patient went to sleep. In thirty minutes more I succeeded in reducing the hernia with the greatest ease. He was thoroughly narcotised, and the muscular system perfectly relaxed. We gave strong coffee freely, moved bowels by enema, and in sixteen hours he was able to be up. He complained of but little soreness or inconvenience about the ring, notwithstanding the tumor had been "worked with" frequently and sometimes severely, for the last sixty-five hours.

Convalescence was rapid, and in four days he resumed business.

We are aware that the number of cases here reported are insufficient to establish any one principle in the practice of medicine. But the report contains all the cases of strangulated hernia that have occurred in my range of practice since 1850. The result of the treatment we deem sufficient apology for presenting the subject for your consideration. Hoping the members will give it a candid consideration, and a fair trial when opportunity offers. If my conclusions are correct, that opium prevents inflammation and gangrene, you are running no risk, for if you fail with this treatment you can still resort to the knife, and with less danger of the operation being followed by a fatal peritonitis.

There may be nothing essentially new in this mode of treatment, but if it has ever been recommended or practiced in this country I am in "blissful ignorance" on the subject. It certainly is not spoken of in our text books or in any medical literature that has come under my observation.

—

Since the above paper was read the opium treatment has been tried by several members of our society. Dr. A. H. Luce, of Bloomington, reports two cases treated in this way with entire success, and Dr. Harrison Noble, of Keyworth, one. Dr. I. W. Coleman, of Le Roy, has furnished me with a full report of a case treated by himself and Dr. T. D. Fisher, an abstract of which I furnish for publication as a continuation of the essay above.

Called April 1st, 1859, at 3 o'clock P. M., to see Mr. A. C., age 55, whose hernia had become strangulated the evening before. Found him suffering much pain, with retching and vomiting of stercoraceous matter. I placed him in situation, and endeavored to reduce it by taxis, but failed, after repeated trials. Having been associated with Dr. S. W. Noble, in the treatment of one of the cases reported in his paper, I at once resolved to resort to the opium treatment, having faith that it was equal to the emergency. Prescribed opii. gr. i, every hour, until he should become narcotised, and left, promising to visit him early next morning.

Being unavoidably detained, I did not get to see him till noon of the next day. Learned that patient had slept soundly during the night, and on waking at six in the morning, much to his joy, his hernia had returned. But having occasion to pass his water, while standing over the chamber, his hernia came down again. The pain and vomiting having returned, we a second time put him on the opium, and left him. Next morning, at day-break, we were summoned in haste to see him, the messenger reporting him decidedly worse. Dr. T. D. Fisher, his family physician, having been absent and returned, visited the patient with me. Patient was much exhausted from loss of sleep, pain, and vomiting, and on inquiry we learned that the friends, after giving him one of the powders and his throwing that up, concluded that it was the medicine which caused so much irritability of the stomach, and did not give any more.

After consultation we concluded to give morphine, gr. i, and wait awhile, and then try the taxis; but did not succeed in the reduction of the tumor. Cold water, ether, and enemas were tried, with no better result.

Not yet discouraged, we decided to give the opium a fourth and last trial. Prescribed morphine, gr. i, every two hours, until narcotism came on, and left, promising to be back in the evening. On our return we found the patient comfortable; vomiting had ceased, and the tumor was materially reduced in size. The bowels having been moved, taxis was again applied, and the hernia was replaced without difficulty. Convalescence was rapid.

Le Roy, Ill., Feb. 13, 1860.

